# Supplementary material for: Study on air temperature estimation and its influencing factors in a complex mountainous area
Source: PLoS One. 2022 Aug 16;17(8):e0272946. doi: 10.1371/journal.pone.0272946 (PMC9380917; doi:10.1371/journal.pone.0272946)
Supplement: Attachment — Submitted filename: Response to Reviewers2-(PONE-D-22-09843).docx [file pone.0272946.s001.docx]

**Response to Reviewers**

Reviewer #2:

The idea and practice of applying (1) remote sensing data like MODIS LST, emissivity, and NDVI, (2) gridded products like land cover and land use, and dem, (3) observation data to build near surface air temperature dataset through the BP-ANN algorithm in Tianshui during 2001~2020. This work lacks scientific merit and the manuscript does not make any significant improvement to the intended field of research.

**Reply:** Thank you very much

(1) It is very important to accurately obtain the spatial and temporal distribution of near-surface temperature for better understanding of land surface processes and drought monitoring at regional scale.

(2) MODIS data has a long time series, which can be used as input data to estimate temperature and reflect the trend of temperature change in the past 20 years. In the study, we reconstructed the MODIS remote sensing data with poor quality by interpolation, which ensured the continuity of the data more than before, when only selecting MODIS data with good quality. Land cover, land use and DEM were used as the data required to drive the model.

(3) BP-ANN algorithm was used to estimate air temperature by referring to previous methods, and its influencing factors were discussed. The research aims to reveal the spatio-temporal changes of air temperature in complex mountainous areas under the background of global temperature changes, and the research results provide a reference for drought monitoring in this region.
