# Supplementary material for: Study on air temperature estimation and its influencing factors in a complex mountainous area
Source: PLoS One. 2022 Aug 16;17(8):e0272946. doi: 10.1371/journal.pone.0272946 (PMC9380917; doi:10.1371/journal.pone.0272946)
Supplement: Attachment — Submitted filename: Response to Reviewers.docx [file pone.0272946.s002.docx]

**Response to Reviewers**

Reviewer :

While revising your submission, please upload your figure files to the Preflight Analysis and Conversion Engine (PACE) digital diagnostic tool, https://pacev2.apexcovantage.com/. PACE helps ensure that figures meet PLOS requirements. To use PACE, you must first register as a user. Registration is free. Then, login and navigate to the UPLOAD tab, where you will find detailed instructions on how to use the tool. If you encounter any issues or have any questions when using PACE, please email PLOS at figures@plos.org. Please note that Supporting Information files do not need this step.

**Reply:** Thank you very much

We have already uploaded the figure files to the Preflight Analysis and Conversion Engine (PACE) digital diagnostic tool, and all the figures have passed the inspection and meet PLOS requirements.
